# The potential neuroprotective role of a histone deacetylase inhibitor, sodium butyrate, after neonatal hypoxia-ischemia

**DOI:** 10.1186/s12974-017-0807-8

**Published:** 2017-02-10

**Authors:** Joanna Jaworska, Malgorzata Ziemka-Nalecz, Joanna Sypecka, Teresa Zalewska

**Affiliations:** NeuroRepair Department, Mossakowski Medical Research Centre, Polish Academy of Sciences, 5 A. Pawinskiego Street, 02-106 Warsaw, Poland

**Keywords:** Neonatal hypoxia-ischemia, Histone deacetylase inhibitors, Sodium butyrate, Neuroprotection, Microglia, Astrocytes, Cytokines, Inflammation

## Abstract

**Background:**

Histone deacetylase inhibitor (HDACi), sodium butyrate (SB), has been shown to be neuroprotective in adult brain injury models. Potential explanation for the inhibitor action involves among others reduced inflammation. We therefore anticipated that SB will provide a suitable option for brain injury in immature animals. The aim of our study was to test the hypothesis that one of the mechanisms of protection afforded by SB after neonatal hypoxia-ischemia is associated with anti-inflammatory action. We examined the effect of SB on the production of inflammatory factors including analysis of the microglial and astrocytic cell response. We also examined the effect of SB on molecular mediators that are crucial for inducing cerebral damage after ischemia (transcription factors, HSP70, as well as pro- and anti-apoptotic proteins).

**Methods:**

Seven-day-old rat pups were subjected to unilateral carotid artery ligation followed by 60 min of hypoxia (7.6% O_2_). SB (300 mg/kg) was administered in a 5-day regime with the first injection given immediately after hypoxic exposure. The damage of the ipsilateral hemisphere was evaluated by hematoxylin-eosin staining (HE) 6 days after the insult. Samples were collected at 24 and 48 h and 6 days. Effects of SB on hypoxia-ischemia (HI)-induced inflammation (cytokines and chemokine) were assessed by Luminex assay and immunohistochemistry. Expression of molecular mediators (NFκB, p53, HSP70, COX-2, pro- and anti-apoptotic factors Bax, Bcl-2, caspase-3) were assayed by Western blot analysis.

**Results:**

SB treatment-reduced brain damage, as assessed by HE staining, suppressed the production of inflammatory markers—IL-1β, chemokine CXCL10, and blocked ischemia-elicited upregulation of COX-2 in the damaged ipsilateral hemisphere. Furthermore, administration of SB promoted the conversion of microglia phenotype from inflammatory M1 to anti-inflammatory M2. None of the investigated molecular mediators that are known to be affected by HDACis in adults were modified after SB administration.

**Conclusions:**

Administration of SB is neuroprotective in neonatal hypoxia-ischemia injury. This neuroprotective activity prevented the delayed rise in chemokine CXCL10, IL-1β, and COX-2 in the ipsilateral hemisphere. SB appears to exert a beneficial effect via suppression of HI-induced cerebral inflammation.

## Background

Histone deacetylase inhibitors (HDACis) are a heterogeneous group of agents that inhibit histone deacetylases (HDACs) and promote posttranslational acetylation of lysine residues within nuclear and cytoplasmic proteins, which may alter their activity and function. In particular, HDAC inhibition can have a profound effect on the acetylation status of histone proteins within chromatin, resulting in the augmented expression of genes relevant to protection from an ischemic insult. In addition, inhibition of deacetylation equally promotes the acetylation of non-histone proteins, such as transcription factors, signal transduction mediators, determining their interaction, localization, and stability [1]. It is very likely that the non-specificity of deacetylase inhibitors is responsible for the opposing effect noted in distinct type of cells. As it is becoming apparent, HDAC inhibition promotes the demise of tumor cells. The same drugs display strong protective properties for neurons in in vitro and in vivo models of neurotoxicity and neurodegeneration (for rev see [2]).

Furthermore, it was reported recently that the treatment of adult animals with histone deacetylase inhibitors, such as trichostatin A (TSA), sodium butyrate (SB), and vorinostat (SAHA), administered just before as well as after the onset of stroke, provides neuroprotection [3–8]. The neuroprotective effect of these agents has been associated with decreasing the lesion volume, neurobehavioral improvement, and stimulation of neurogenesis in the ischemic adult brain [9, 10]. Despite the growing number of evidence supporting the beneficial effect of HDACis in the experimental model of stroke in adult rodents, only a few available reports were addressed upon their effect in the hypoxia-ischemia (HI)-injured immature brain [11–13]. However, due to different experimental paradigms, it is not possible to make the explicit conclusion.

Neonatal HI encephalopathy still remains one of the most important causes of neonatal mortality and/or long-term neurological sequelae such as cerebral palsy, seizure disorders, cognitive and intellectual deficits, and behavioral problems [14–17]. Currently, there are no well-established treatments to reduce brain damage and it is still a big challenge to protect the newborns’ brain from HI injury. The only available effective treatment, hypothermia, neither provides complete brain protection nor stimulates the repair necessary for neurodevelopmental outcome. Recently, HDACis are being considered as valuable tools to reduce or even to prevent HI-induced brain damage in neonates. Since many aspects of the evolving brain damage following the insult differ between adults and neonates, extrapolating data obtained in the mature brain to neonates is generally unwise. Therefore, the present study was undertaken to examine whether treatment with one of the HDACis, sodium butyrate (SB), has neuroprotective effects in a rat model of neonatal HI. We aimed to assess whether SB action is associated with changes in molecular mediators that are crucial for inducing cerebral damage and thus be targeted for therapy. As inflammation is a well-recognized pathogenic factor in perinatal brain injury, we analyzed the microglial and astrocytic cell response to SB treatment and the influence of SB on cytokines, transcription factors, HSP70, and pro- and anti-apoptotic proteins.

## Methods

Experimental animal work was conducted according to regulations following European Union directives. Experimental procedures were approved by the Local Ethics Committee for Animal Experimentation. All efforts were made to minimize the number of animals and animal suffering in every step.

### Experimental neonatal hypoxia-ischemia

Animals were housed under controlled temperature (22 °C ± 2), with a 12-h light cycle period and pelleted food and water ad libitum. Cerebral hypoxia-ischemia was produced in 7-day-old (P7) Wistar rats of either sex by a permanent unilateral common carotid artery ligation, followed by systemic hypoxia [18, 19]. As was previously reported, the ligation alone does not decrease cerebral perfusion below critical levels and the addition of hypoxia is required to cause brain infarct [20]. Briefly, pups were anesthetized with isoflurane (4% induction, 2% maintenance) carried by O_2_. Once they were fully anesthetized, a midline neck incision was made and the left common carotid artery was exposed, double ligated with surgical silk, and cut between two ligatures. The incision was then sutured with monofilament nylon. Sham-operated animals underwent the same surgical procedure without the ligation of the carotid artery. The time length of anesthesia lasted on average 5 min. After surgery, the rat pups were returned to their home cage for 1 h to recover. Later, the animals were placed for 1 h in a hypoxic chamber containing 7.6% oxygen balanced with nitrogen with controlled humidity and temperature maintained at 35 °C.

The undamaged hypoxic hemisphere, as well as age-matched sham-operated animals, served as controls. Pups from each litter were randomly assigned to four experimental groups (5 rats per group): (1) control group (vehicle treatment), (2) control animals (SB treatment), (3) animals which underwent HI (vehicle treatment), and (4) animals which underwent HI (SB treatment). Animals were sacrificed at specific time points (12, 24, 48, 72 h and 6 days) after the injury.

### Drug administration

Rats subjected to HI or sham operated were treated once a day with subcutaneous injections of sodium butyrate (SB; Sigma-Aldrich; 300 mg/kg body wt) [4] or vehicle (saline) starting immediately after hypoxic exposure and lasting up to 5 consecutive days.

### Tissue preparation

Six days after HI-anesthetized animals were perfused transcardially first with phosphate-buffered saline (PBS) followed by a fixative solution (4% paraformaldehyde, PFA, in 0.1 M phosphate buffer, pH 7.4). The brains were removed and submerged in the same fixative solution for 4 h at 4 °C. Following postfixation brains were cryoprotected overnight in 30% sucrose solution (in 0.1 M PBS), frozen rapidly using dry ice, and placed in −80 °C storage.

For biochemical analysis, animals were sacrificed (12, 24, 48, 72h and 6 days after HI) through decapitation and the whole hemispheres were frozen on dry ice. All tissue samples were stored at −80 °C until used.

### Brain injury evaluation

Hematoxylin-eosin (HE) staining was performed to evaluate the neuroprotective effect of SB against ischemia-induced brain damage. Six days after the insult (at postnatal day 13), the pups were anesthetized with 100 mg/kg ketamine combined with 10 mg/kg xylazine and perfused. The brains were dissected and frozen on dry ice. Coronal cryostat sections (20 μm) were stained with HE and examined using light microscopy.

### Immunohistochemistry

The following antibodies (source and final dilution) were used for tissue staining: mouse monoclonal anti-ED1 (CD68) (AbD Serotec, 1:100), goat polyclonal anti-Arg-1 (arginase-1) (Santa Cruz, 1:250), rabbit polyclonal anti-IL-1β (Santa Cruz, 1:250), rabbit polyclonal anti-GFAP (Glial Fibrillary Acidic Protein, DAKO, 1:200), and chicken polyclonal anti-GFAP (Millipore, 1:200).

Coronal cryostat sections of the brain (30 μm thick) were cut at the level of the lateral ventricles in serial order to create 10 series sections. Double fluorescent immunohistochemistry was performed on free-floating sections. After blocking for unspecific reactivity, adjacent series of sections were stained for a specific cell-lineage marker.

For identification of the type of microglia, we used markers labeling M1 (ED1/IL-1β) and M2 (ED1/arginase-1) cells. Double labeling was also employed for monitoring astrocytes expressing IL-1β. Tissue sections were rinsed in PBS and then incubated in 10% normal goat serum in PBS containing 0.25% Triton X-100 for 60 min in room temperature (RT). Next, the sections were washed with PBS and incubated with anti-ED-1 or anti-GFAP overnight at 4 °C. The following day, tissue sections underwent the washing procedure, and the primary antibodies were revealed by applying appropriate secondary FITC-conjugated antibodies (AlexaFluor, 1:500) for 60 min at room temperature and in the dark. After this step, the sections were rinsed in PBS and incubated with primary antibodies (anti-Arg-1 or anti-IL-1β) overnight at 4 °C. The next day, after being rinsed in PBS, the sections were exposed to appropriate Cy3-conjugated secondary antibodies (AlexaFluor, 1:500) for 1 h at room temperature. Nuclei were subsequently labeled with the fluorescent dye Hoechst 33258 (2 μg/ml PBS; Sigma).

Labeling was verified using a confocal laser scanning microscope (LSM 780, Carl Zeiss, Germany) using a 10× or 20× objective. A helium-neon laser (543 nm) was utilized in the excitation of Alexa Fluor 546, while an argon laser (488 nm) was applied in the excitation of FITC. ImageJ software was used for quantitative analysis of immunoreactive sections. Five animals per group were analyzed. Images from five sections per animal were taken, and the number of positive-labeled cells as well as fluorescence intensity was assessed in an area of 1.44 mm^2^.

### Determination of cytokine expression in brain extracts

Concentrations of chemokines/cytokines were measured in extracts from brain hemispheres using the EMD Millipore’s MILLIPLEX® MAP Rat Cytokine/Chemokine Magnetic Bead assay according to the manufacturer’s instructions. The cytokines and chemokines analyzed included TNFα, IL-1α, IL-1β, IL-2, IL-4, IL-6, IL-12, IFN-γ, and chemokine CXCL10 (IP-10). The median fluorescence intensity plates were assayed on a Bio-Plex® 200 Luminex system with Bio-Plex Manager 5.0 software. The five-parameter logistic method was applied to estimate cytokine/chemokine concentrations in brain homogenates.

### Quantitative polymerase chain reaction (real-time PCR)

Gene expression of pro-inflammatory (TNFα, IL-1β) cytokines was evaluated in the brain hemispheres obtained from rats 12, 48 and 72 h after HI. Total RNA was isolated with TRIzol Reagent, and the quality and concentration of RNA was verified by spectrophotometry with the Nanodrop™ apparatus. The samples containing 1 μg of total RNA were reverse transcripted using High Capacity RNA-to-cDNA Kit (Applied Biosystems) according to the manufacturer’s instructions.

Quantitative real-time PCR analyses of cDNA samples (300 ng) with designed specific primers (Table 1) and Fast SYBR Green Master Mix (Applied Biosystems) were performed in 7500 Fast Real-Time PCR System (Applied Biosystem). Reaction parameters were as follows: (1) holding stage, 20 s at 95 °C; (2) cycling stage (40×), 3 s at 95 °C and 30 s at 60 °C; and (3) melt curve stage, 15 s at 95 °C, 1 min at 60 °C, 15 s at 95 °C, and 15 s at 60 °C. Each sample was tested in triplicate during two analyzing sessions. The fluorescence signal from specific transcript was normalized against that of reference gene (SDHA), and threshold cycle values (ΔCt) were quantified as fold changes by the 2^−ΔΔCT^ method.

**Table 1:** List of designed primers used in reverse transcription and quantitative real-time (RT)-PCR analysis

Gene	Forward primer sequence	Reverse primer sequence
IL-1β	5′-CACCTCTCAAGCAGAGCACAG-3′	5′-GGGTTGCATGGTGAAGTCAAC-3′
TNFα	5′-AAATGGGCTCCCTCTCATCAGTCC-3′	5′-TCTGCTTGGTGGTTTGCTACGAC-3′
SDHA	5′-CCCTGAGCATTGCAGAATC-3′	5′-CATTTGCCTTAATCGGAGGA-3′

### Western blot analysis

The following antibodies (source and final dilution) were used for analysis: mouse monoclonal anti-NFκB (Cell Signaling, 1:1000), rabbit polyclonal anti-p53 (Cell Signaling, 1:1000), rabbit polyclonal anti-HSP70 (Cell Signaling, 1:1000), rabbit monoclonal anti-COX-2 (Cell Signaling, 1:1000), rabbit polyclonal anti-Bcl-2 (Cell Signaling, 1:1000), rabbit polyclonal anti-Bax (Cell Signaling, 1:1000), and mouse monoclonal anti-actin (MP Biomedicals, 1:500).

Brain tissues were homogenized in RIPA lysis buffer (10 mM Tris-HCl pH 7.5 containing 150 mM NaCl, 1% Nonidet P40, 0.1% SDS, 1% Triton X-100, PMSF 0.1 mg/ml) and a proteinase and phosphatase inhibitor cocktail (Life Technologies, 1:100). Lysates were clarified by centrifugation at 13000 *g* for 10 min at 4 °C. The supernatant was collected, and protein concentrations were determined using a Bio-Rad DCTM protein assay kit (Bio-Rad). Samples (50 μg protein) were ran on 10–15% SDS-PAGE gels and transferred onto nitrocellulose membranes (Amersham Bioscience). After blocking, membranes were probed with specific primary antibodies and then incubated with horseradish peroxidase-conjugated secondary IgG antibodies (Sigma-Aldrich). Immunoblot signals were visualized using ECL chemiluminescence kit (GE Healthcare Life Sciences). To verify an equal loading of protein per line, the β-actin antibody was used as an internal control for each immunoblotting. Semi-quantitative evaluation of protein levels detected by immunoblotting was performed by computer-assisted densitometric scanning (LKB Utrascan XL, Program GelScan). The level of protein immunoreactivity was determined by frequent analysis of multiple immunoblots.

### Quantitative measurement of prostaglandin E2 protein concentration

To estimate the amount of prostaglandin E2 (PGE2) in homogenates obtained from the brain hemispheres, the Prostaglandin E2 ELISA Kit-Monoclonal (Cayman) test was applied according to the supplier’s instructions. Frozen hemispheres were homogenized in 1 ml of 0.1 M phosphate buffer (pH 7.4) containing 1 mM EDTA and 10 μM indomethacin. Homogenates were clarified by centrifugation at 8000 *g* for 10 min at 4 °C, and the supernatant was collected for analysis. Protein concentrations were determined using a Bio-Rad DC™ protein assay kit (Bio-Rad). After performing the Sandwich ELISA assay, the plates were read at 412 nm using a spectrophotometric plate reader Fluorostar Omega (BMG LabTech).

### Quantitative measurement of caspase-3 activity

To estimate the level of activated caspase-3 in lysates obtained from both brain hemispheres, the Caspase-3 Fluorescence Assay Kit (Cayman Chemical) was applied according to the supplier’s instructions. Briefly, the kit employs a specific caspase-3 substrate, N-Ac-DEVD-N'-MC-R110, which, upon cleavage by active caspase-3, generates a highly fluorescent product that is easily quantified. The fluorescence intensity of each was well read using a spectrophotometric plate reader Fluorostar Omega (BMG LabTech; excitation = 485 nm, emission = 535 nm).

### Statistical analysis

GraphPad PRISM 5.0 software was used for the statistical analysis of the received data. Comparisons between animal groups were performed using the one-way analysis of variance (ANOVA) followed by the Bonferroni post-hoc test for multiple comparisons or Student’s *t* test. All values are expressed as mean ± SD. The data were considered significant at *p* value <0.05.

## Results

### Sodium butyrate reduces brain damage after neonatal HI

**Fig. 1:**
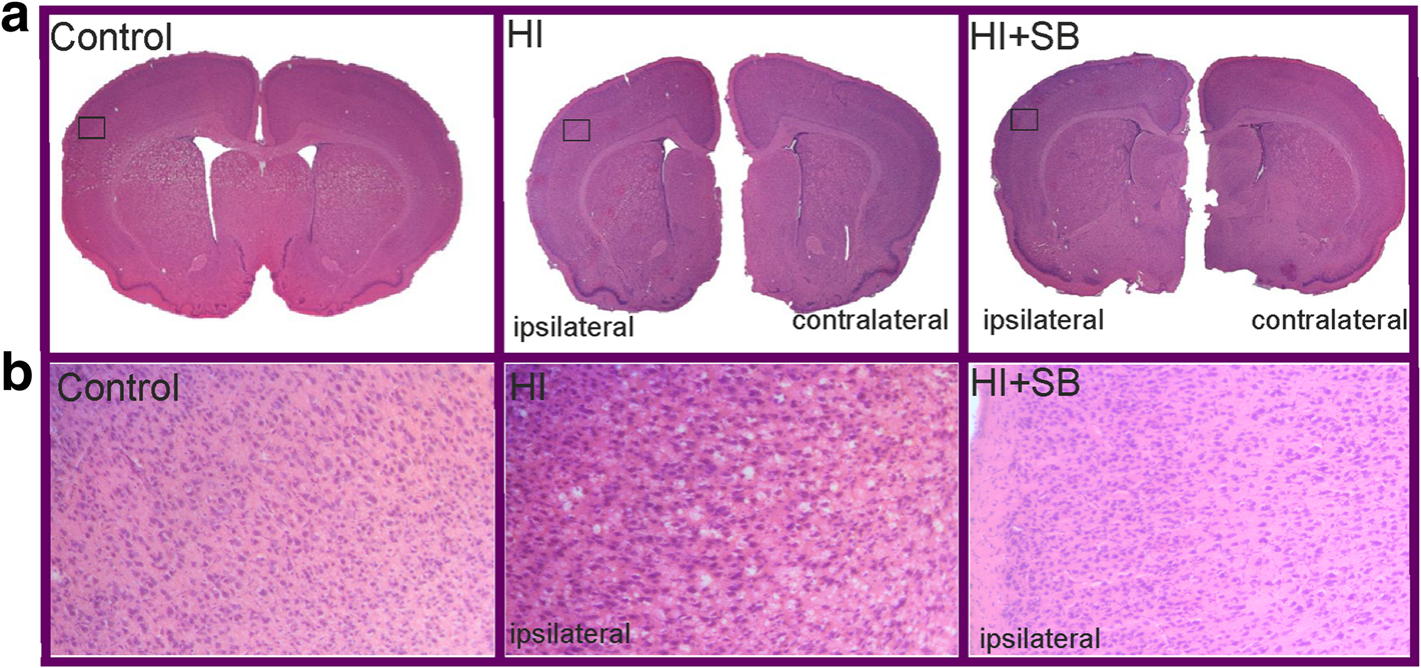
Sodium butyrate treatment reduces hypoxia-ischemia- induced brain damage in neonates. Seven-day-old rats (PND7) were subjected to hypoxia-ischemia followed by 6 days of recovery. SB or vehicle was administered directly after the onset of HI and for 5 consecutive days. **a** Brain coronal sections from sham control animals and from animals 6 days after hypoxia-ischemia (with or without SB treatment) were stained with hematoxylin-eosin (HE). **b** Lower panel represents magnification (100×) of the ipsilateral hemisphere area (marked with rectangles in **a**). Note the loss of neurons and signs of cerebral edema in the cortex of ipsilateral hemisphere. Sodium butyrate administration provided almost complete neuroprotection in comparison with non-treated animals. Photomicrographs are representative of observations made from five animals per group

Both the left and right brain hemispheres of all rats (sham control, HI with or without SB treatment) were subjected to histological evaluation at 6 days after the insult (P13). Coronal sections (cut at the level of the lateral ventricles) stained with HE show the loss of neurons and signs of cerebral edema with swollen cells throughout the ipsilateral frontal cortex exclusively (Fig. 1). Administration of SB immediately after HI provided almost complete neuroprotection in comparison with non-treated animals. Neither neuronal loss nor edema was observed. Furthermore, the brain slices demonstrated proper cytoarchitecture.

### SB-modified microglial/macrophage and astroglial response to neonatal HI

#### Microglia

**Fig. 2:**
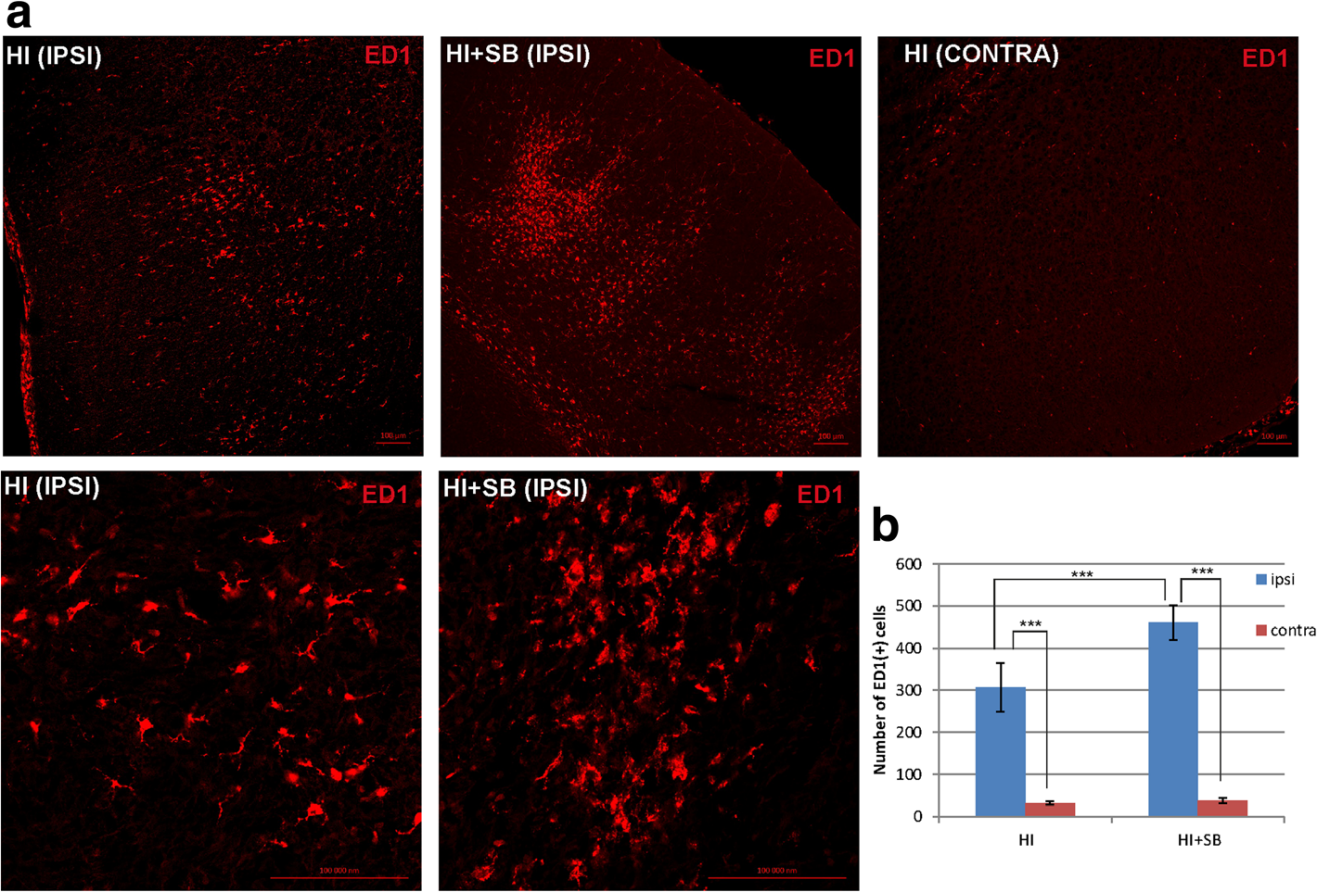
Sodium butyrate increases microglial/macrophage cell number in the ipsilateral hemisphere after neonatal hypoxia-ischemia. Seven-day-old rats (PND7) were subjected to hypoxia-ischemia followed by 6 days of recovery. SB or vehicle was administered directly after the onset of HI and for 5 consecutive days. Brain sections were stained for ED1 immunoreactivity (*red*). **a** Confocal photomicrographs show immunohistochemical reaction in the frontal cortex of ipsilateral (injured) and contralateral (control) hemispheres with or without SB treatment. Numerous ED1-positive cells are mainly seen in ipsilateral side, and their number further increases after SB administration. Lower panel represents magnification of the upper ipsilateral photomicrographs. Scale bar 100 μm. **b** Graph shows the number of ED1-labeled cells quantified in the frontal cortex (1.44 mm^2^ area). The values represent means ± SD of five animals per each experimental group. The one-way ANOVA and Bonferroni test indicate significant differences in the number of ED1(+) cells between the investigated groups: ***p < 0.001. *IPSI* ipsilateral hemisphere, *CONTRA* contralateral hemisphere

To determine the effect of SB administration on cerebral activation/influx of microglia/macrophages after hypoxia-ischemia, we performed ED1 staining on the brain sections of sham-operated, HI, and HI + SB rat pups. The data presented in Fig. 2 shows numerous ED1-positive cells in ipsilateral hemisphere at 6 days after HI. Most microglial cells were round shaped with thick processes and were considered to be in an activated state. The activated microglial cells were scattered throughout the entire cortex and striatum. Contrary, in slices obtained from control animals, as well as from contralateral hemispheres, the activated microglial cells were not detected (*p* < 0.001, ipsi vs. contra). Sodium butyrate administration resulted in an increased number of microglial cells to 150% of vehicle-treated animals in the ipsilateral side.

**Fig. 3:**
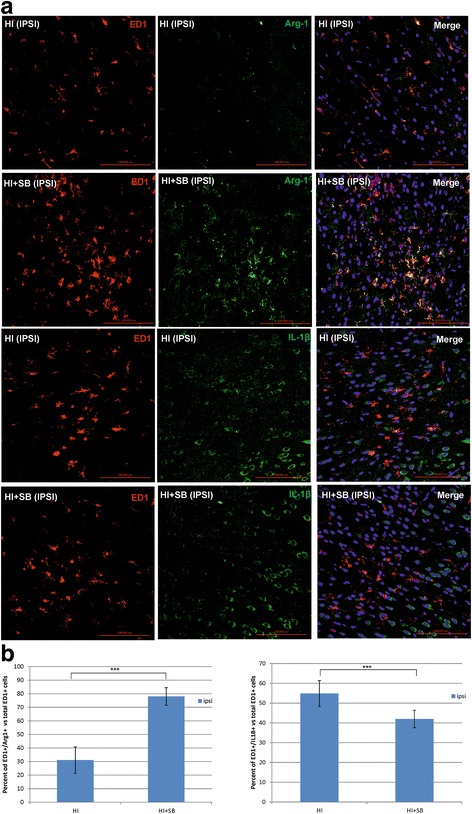
Sodium butyrate promotes the polarization of microglia from M1- to M2-like phenotype after neonatal hypoxia-ischemia. Seven-day-old rats (PND7) were subjected to hypoxia-ischemia followed by 6 days of recovery. SB or vehicle was administered directly after the onset of HI and for 5 consecutive days. Sections from ipsilateral hemispheres were stained for ED1 immunoreactivity (*red*), for arginase-1 (Arg-1), marker specific for M2 phenotype (*green*), and for IL-1β, marker for M1 phenotype (*green*). Nuclei were labeled with the Hoechst dye (*blue*). Six days after HI, the majority of ED1-positive cells expressed IL-1β, with only a few cells co-stained with ED1/Arg-1. The administration of SB after HI led to a marked decrease in the amount of cells presenting the M1 (ED1/IL-1β positive) phenotype of microglia with concomitant enhancement of cells stained with ED1/Arg-1 specific for M2 type. **a** Photomicrographs are representative of observations made from 5 animals per experimental group. Scale bar 100 μm. **b** Graphs show the percent of the ED1(+)/Arg-1(+) and ED1(+)/IL-1β (+) cells versus total pool of ED1-positive cells quantified in the frontal cortex (1.44 mm^2^ area). The values represent means ± SD of five animals per each experimental group. Student’s *t* test indicates significant differences in the number of ED1(+)/Arg-1(+) and ED1(+)/IL-1β (+) cells between the investigated groups: ***p < 0.001. *IPSI* ipsilateral

Next, we examined whether SB promotes the polarization of microglia from M1- to M2-like phenotype after HI. To address this, we performed double staining with IL-1β antibody coupled with ED1 for the identification of activated proinflammatory M1 phenotype and ED1/arginase-1 for anti-inflammatory M2-like phenotype (Fig. 3a, b). Six days after HI, the majority of ED1-positive cells expressed IL-1β in the cortical region of the ipsilateral hemisphere, with only a few cells stained positively with ED1/Arg-1. The administration of SB after HI led to a marked decrease in the amount of cells presenting the M1 phenotype of microglia (HI vs HI + SB, *p* < 0.001) with concomitant enhancement of cells stained with ED1/Arg-1 specific for M2 type (HI vs HI + SB, *p* < 0.001).

#### Astrocytes

**Fig. 4:**
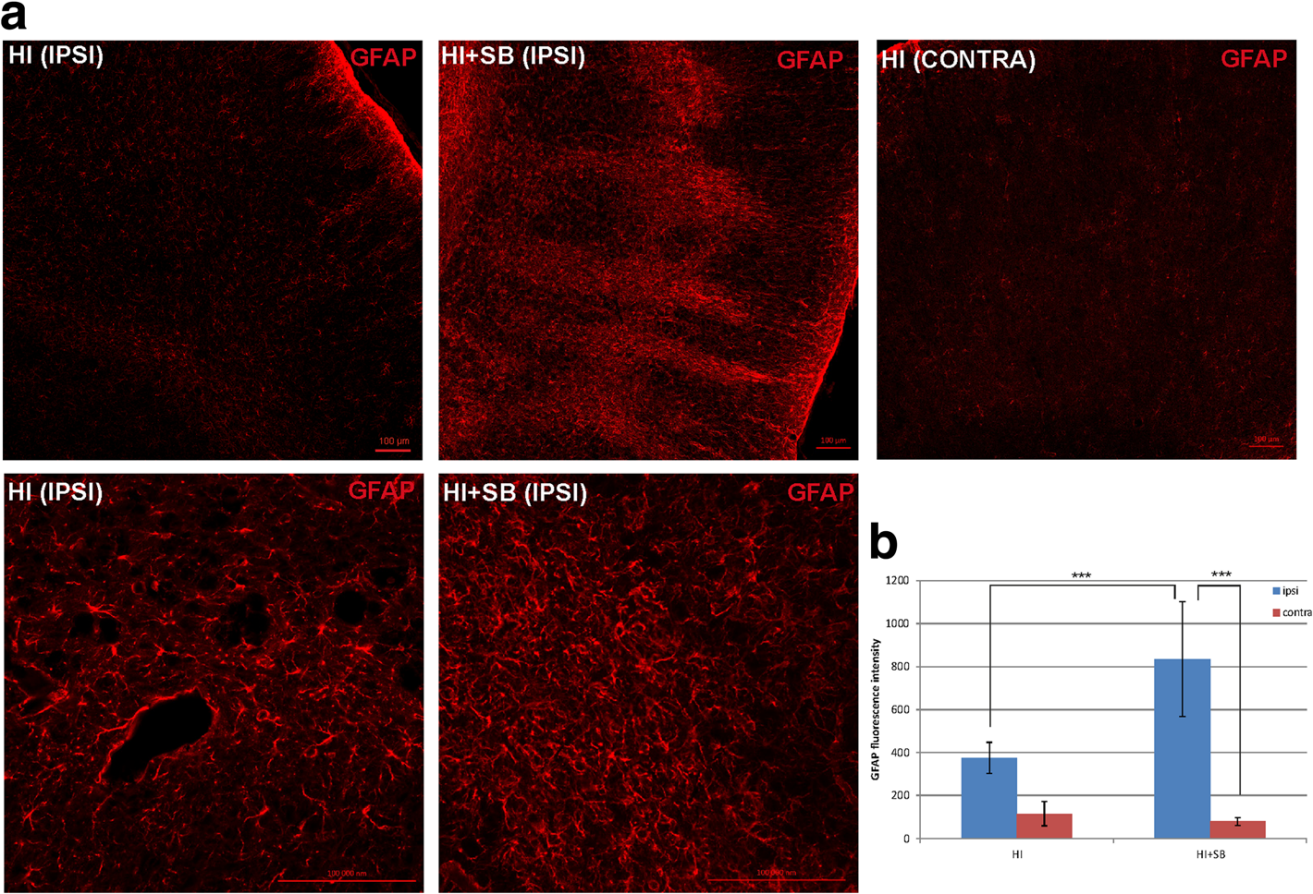
Sodium butyrate increased the amount of reactive astrocytes in the rat ipsilateral hemisphere after neonatal hypoxia-ischemia. Seven-day-old rats (PND7) were subjected to hypoxia-ischemia followed by 6 days of recovery. SB or vehicle was administered directly after the onset of HI and for 5 consecutive days. Brain sections were stained for GFAP immunoreactivity (*red*). **a** Confocal photomicrographs show immunohistochemical reaction in the frontal cortex of contralateral (control) and ipsilateral (injured) hemispheres with or without SB treatment. Note the increased amount of GFAP-positive cells in injured side 6 days after hypoxia-ischemia. SB administration resulted in a significant elevation in the GFAP staining intensity in the ipsilateral hemisphere in comparison to vehicle-treated animals. Lower panel represents magnification of the upper photomicrographs. Scale bar 100 μm. **b** Graph shows the GFAP-associated fluorescent signal quantified in the frontal cortex (1.44 mm^2^ area). The values represent means ± SD of five animals per experimental group. The one-way ANOVA and Bonferroni test indicate significant differences in GFAP fluorescence intensity between the investigated groups: ***p < 0.001. *IPSI* ipsilateral, *CONTRA* contralateral

Figure 4 shows that 6 days after neonatal HI, the GFAP-associated fluorescent signal increased in the ipsilateral hemisphere; however, this elevation was not statistically significant compared to the contralateral side. Astrocytes presented an activated phenotype characterized by hypertrophic processes. SB treatment resulted in an over twofold elevation in the GFAP staining intensity in the ipsilateral hemisphere. In addition, the hypertrophy of astrocytic cells was more pronounced and associated with inter-digitations of processes that overlapped and formed glial scars.

**Fig. 5:**
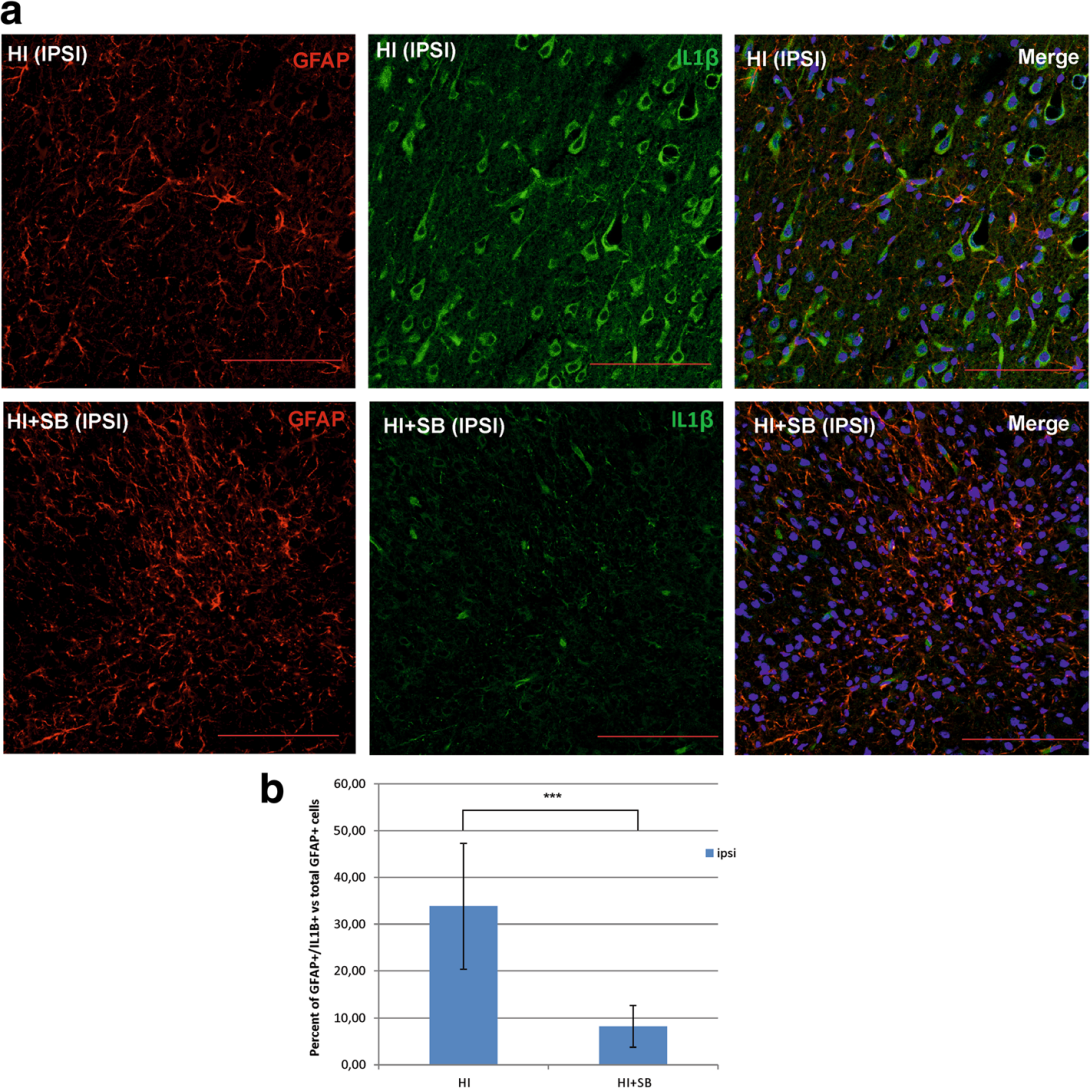
Sodium butyrate decreases IL-1β expression in astrocytes. Seven-day-old rats (PND7) were subjected to hypoxia-ischemia followed by 6 days of recovery. SB or vehicle was administered directly after the onset of HI and for 5 consecutive days. Brain sections from ipsilateral hemispheres were stained for GFAP immunoreactivity (*red*) and for IL-1β (*green*). Nuclei were labeled with the Hoechst dye (*blue*). The majority of astroglial cells expressed IL-1β within the cortex of ipsilateral side. The amount of these cells was markedly reduced after SB administration. **a** Photomicrographs are representative of observations made from 5 animals per experimental group. Scale bar 100 μm. **b** Graph shows the percent of the GFAP(+)/IL-1β (+) cells versus total pool of GFAP-positive cells quantified in the frontal cortex (1.44 mm^2^ area). The values represent means ± SD of five animals per each experimental group. Student’s *t* test indicates significant differences in the number of GFAP(+)/IL-1β (+) cells between the investigated groups: ***p < 0.001. *IPSI* ipsilateral

We also determined the effect of SB treatment after HI on the number of GFAP-positive cells co-stained with cytokine IL-1β antibody (Fig. 5a, b). Our results show that 6 days following HI, a majority of astroglial cells express IL-1β within the cortex of the damaged ipsilateral hemisphere. The amount of these cells was markedly reduced upon SB administration (HI vs HI + SB, *p* < 0.001).

### Effect of SB on inflammatory markers

#### Effect of SB on cytokines

Cytokines IL-1α, IL-1β, IL-2, IL-4, IL-6, IL-12, chemokine CXCL-10, tumor necrosis factor alpha (TNFα), and interferon-gamma (IFN-γ) were measured, and differences between ipsilateral (hypoxic-ischemic) vs contralateral (hypoxic) hemispheres, as well as vs sham control, were compared at 48 h and 6 days after the injury in animals treated with vehicle or SB.

**Fig. 6:**
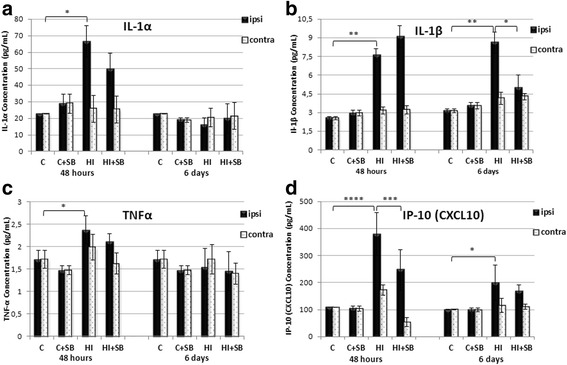
Effects of SB treatment on the level of cytokines/chemokines in the brain after neonatal HI. Seven-day-old rats (PND7) were subjected to hypoxia-ischemia followed by 48 h or 6 days of recovery. SB or vehicle was administered directly after the onset of HI and for 2 or 5 consecutive days (determined by the experimental paradigm). The concentrations of proinflammatory cytokines (pg/ml): IL-1α (**a**), IL-1β (**b**), TNFα (**c**), and chemokine IP-10 (CXCL10) (**d**) were determined in the ipsilateral (injured) and contralateral hemispheres. Bar graphs represent statistical analysis of the data from indicated experimental groups: vehicle control (C), SB-treated control (C + SB), vehicle-treated hypoxia-ischemia (HI), and SB-treated hypoxia-ischemia (HI + SB). Note the suppression of HI-induced upregulation of IP-10 and IL-1β at 48 h and 6 days of recovery, respectively, after SB treatment. The values represent means ± SD from five animals in each group. The one-way ANOVA and Bonferroni test: *p < 0.05, **p < 0.01, ***p < 0.001, and ****p < 0.0001. *C* control, *ipsi* ipsilateral, *contra* contralateral

We found a large individual variation in the cytokine levels after HI, and not all of them present elevated levels after HI (Fig. 6). A significantly increased amount of proinflammatory IL-1α, IL-1β, TNFα, and chemokine CXCL10 (IP-10) was observed in the ipsilateral hemisphere at 48 h compared to the control one. However, the degree of significance in the case of TNFα and IL-1α reached *p* < 0.05, for IL-1β *p* < 0.01, and for the chemokine *p* < 0.0001. It is worth to note that the concentration of IL-1β and chemokine remained elevated up to 6 days of survival time (*p* < 0.01 and *p* < 0.05, respectively, HI vs sham control). In contrast, in the contralateral hemisphere, the level of these molecules did not differ from the controls. SB significantly suppressed upregulation of the CXCL10 chemokine at 48 h post-HI (*p* < 0.001, HI vs HI+SB). The reduction of IL-1β in the HI hemisphere occurred later, at 6 days of recovery (*p* < 0.05, HI vs HI + SB). In the case of TNFα and IL-1α, the SB action was expressed only by the tendency to lessen the concentration of this protein.

No notable differences between hemispheres were observed in IL-2, IL-6, IL-12, IFNγ, and anti-inflammatory IL-4 at 48 h and 6 days post-HI. The expression pattern of these molecules did not change after SB administration (data not shown).

**Fig. 7:**
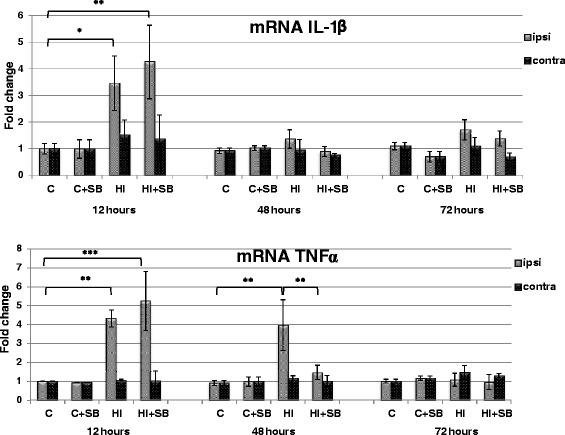
Effects of SB treatment on gene expression of IL-1β and TNFα in the brain after neonatal HI. Seven-day-old rats (PND7) were subjected to hypoxia-ischemia followed by 12, 48, and 72 h of recovery. SB or vehicle was administered directly after the onset of HI and for 2 or 3 consecutive days (determined by the experimental paradigm). The mRNA expression was determined in the ipsilateral (injured) hemispheres as well as in the control brains. Graphs represent statistical analysis of the data from indicated experimental groups: vehicle control (C), SB-treated control (C + SB), vehicle-treated hypoxia-ischemia (HI), and SB-treated hypoxia-ischemia (HI + SB). Note the decrease of HI-induced elevation of TNFα mRNA expression after SB treatment at 48 h of recovery. The values represent means ± SD from five animals in each group. The one-way ANOVA and Bonferroni test: *p < 0.05, **p < 0.01, and ***p < 0.001. *C* control, *ipsi* ipsilateral, *contra* contralateral

To investigate if the pattern of protein concentration changes of IL-1β and TNFα (Fig. 7) is similar to that presented by the expression of their messenger RNA (mRNA), we performed qRT-PCR at different time points following HI. As shown in Fig. 7, HI insult led to a remarkable increase in IL-1β (*p* < 0.05) and TNFα (*p* < 0.01) mRNA level by more than three- and fourfold, respectively, in the ipsilateral hemisphere 12 h after HI, when compared with the matching controls. A significantly elevated level for TNFα mRNA also remained 48 h after HI (*p* < 0.01), but was reduced to the control level in the presence of SB (HI vs HI + SB, *p* < 0.01). This was the only action manifested by the histone deacetylase inhibitor. At a later time point, the level of both cytokines decreased and no considerable changes in gene expression were noticed at 72 h post-HI, regardless of animal groups.

#### Effect of SB on COX-2

**Fig. 8:**
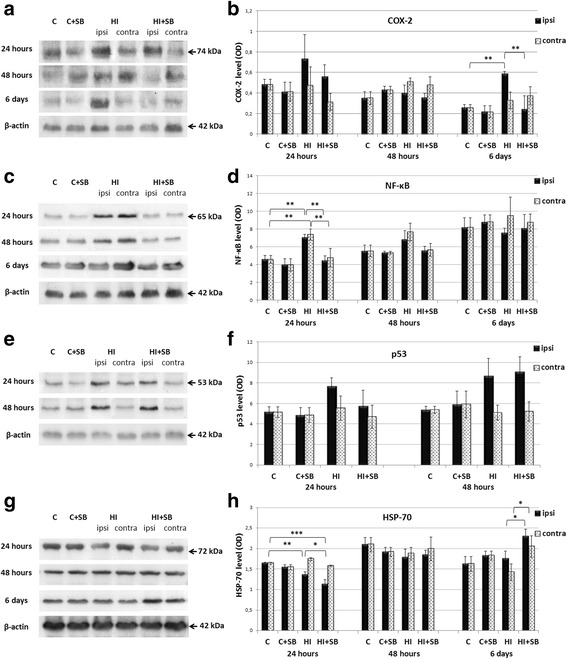
Effects of SB on the expression of COX-2, NfκB, p53, and HSP70 in the brain after neonatal HI. Seven-day-old rats (PND7) were subjected to hypoxia-ischemia followed by 24 and 48 h and 6 days of recovery. SB or vehicle was administered directly after the onset of HI and for 2 or 5 consecutive days (determined by the experimental paradigm). Figure shows representative immunoblots of COX-2 (**a**), NfκB (**c**), p53 (**e**), and HSP-70 (**g**) protein in brain hemispheres, analyzed in four experimental groups: vehicle control (C), SB-treated control (C + SB), vehicle-treated hypoxia-ischemia (HI), and SB-treated hypoxia-ischemia (HI + SB). The intensity of each band obtained by respective Western blotting was quantified by LKB Ultrascan XL software and normalized in relation to β-actin. Bar graphs (**b**, **d**, **f**, **h**) represent statistical analysis of densitometric data (OD units) from indicated experimental groups. Note the decrease of HI-induced elevation of COX-2 immunoreactivity after SB treatment at 6 days of recovery. The administration of SB also resulted in an increased expression of HSP70 protein at the same time point. The values represent means ± SD from five animals. The one-way ANOVA and Bonferroni test: *p < 0.05, **p < 0.01. *C* control, *ipsi* ipsilateral, *contra* contralateral

COX-2 is the rate-limiting enzyme for prostanoid synthesis and an inflammatory marker. The COX-2 protein expression was determined by Western blotting and scanning densitometry (Fig. 8 a, b). At 24 h after HI, we observed only an increasing, however not significant tendency in the hypoxic-ischemic hemisphere. The robust HI-induced elevation of COX-2 protein expression in the ipsilateral hemisphere by about threefolds compared to that of sham control (*p* < 0.01) was seen at 6 days after the insult. Administration of SB reduced the immunoreactivity level to the value presented by the respective sham (*p* < 0.01, HI vs HI + SB).

#### Effect of SB on prostaglandin E_2_

**Fig. 9:**
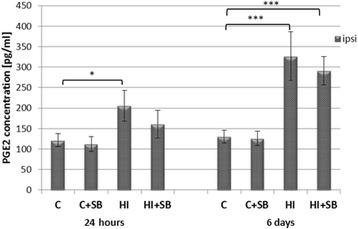
The effect of SB on PGE2 concentration in the brain after neonatal HI. Seven-day-old rats (PND7) were subjected to hypoxia-ischemia followed by 24 h and 6 days of recovery. SB or vehicle was administered directly after the onset of HI and for 5 consecutive days (determined by the experimental paradigm). The PGE2 concentration was determined in the ipsilateral (injured) hemispheres as well as in the control brains. Graphs represent statistical analysis of the data from indicated experimental groups: vehicle control (C), SB-treated control (C + SB), vehicle-treated hypoxia-ischemia (HI), and SB-treated hypoxia-ischemia (HI + SB). SB application had no effect on the HI-induced activation of PGE2 in the HI ipsilateral hemispheres. The values represent means ± SD from five animals in each group. The one-way ANOVA and Bonferroni test: *p < 0.05 and ***p < 0.001. *C* control, *ipsi* ipsilateral

As shown on Fig. 9, the concentration of PGE2 at 24 h after HI markedly increased when compared to control (*p* < 0.05). The administration of SB had no noticeable impact. Furthermore, the pattern of PGE2 changes remains close to that presented by COX2. The elevation of COX-2 protein concentration in the ipsilateral hemisphere seen at 6 days after HI remains in agreement with increased PGE2 (*p* < 0.001). However, despite that treatment with SB induced a decrease in COX-2 expression at this time point, it does not influence the concentration of PGE2 which remains on a high level.

### SB treatment modified expression of transcription factors (NFκB, p53) and HSP70

Subsequently, we checked if SB counteracts the action of chosen transcription factors: NFκB and p53, and HSP70.

#### NFκB

As depicted on Fig. 8 c, d, exposure of 7-day pups to HI caused significant elevation of NFκB, almost equally in the both brain hemispheres (ipsi- and contralateral), compared to the sham control (about 1.5-fold; *p* < 0.01) at 24 h of recovery. As a result of SB treatment, the level of protein returned to the control level and this was the only noticeable effect of the histone deacetylase inhibitor action. At the later time points after the injury, the level of NFκB had the tendency to increase; however, densitometric analysis of the respective blots did not indicate any significant changes between experimental groups.

#### p53

The level of p53, the apoptosis regulating transcription factor, was estimated at 24 and 48 h of recovery after HI. As shown in Fig. 8 e, f, the insult did not alter significantly the immunoreactivity level; however, the trend was noticed towards higher expression of p53 in the hypoxic-ischemic hemisphere, compared with the hypoxic only, contralateral side, as well as with sham control. Importantly, administration of SB after the onset of HI in any case did not suppress the expression level of p53.

#### HSP70

According to generally accepted data indicating the correlation between HSP induction and resistance to brain damage, we aimed to evaluate the role of HSP70 as a potential mediator of the neuroprotective effects of exogenously administered SB. Figure 8 g, h shows representative immunoblots and relative intensity of changes (quantified by scanning densitometry). The data revealed that 24 h after HI, the immunoreactivity of HSP70 declined in the ipsilateral side to 80% of control values (*p* < 0.01, control vs HI). We did not observe any change in HSP70 expression in this time point in the contralateral hypoxic hemisphere of HI-treated rats. Unexpectedly, the administration of SB led to a further decrease of HSP70 expression (to 69% of control; *p* < 0.001). An increased expression of HSP70 after SB injection was found in both brain hemispheres, compared to respective vehicle-treated animals, at 6 days of recovery (*p* < 0.05, vehicle treated vs SB treated).

### Effect of SB on pro- and anti-apoptotic proteins

To address the question whether the neuroprotective action of SB is associated with an influence on apoptosis related factors, we assayed the levels of activated caspase-3, Bax, and Bcl-2.

#### Caspase 3

**Fig. 10:**
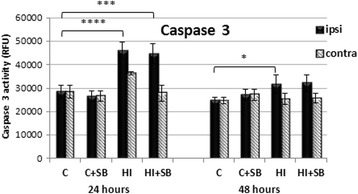
The effect of SB treatment on caspase-3 activity in the brain after neonatal HI. Seven-day-old rats (PND7) were subjected to hypoxia-ischemia followed by 24 or 48 h of recovery. SB or vehicle was administered directly after the onset of HI and for 2 consecutive days. Bar graphs represent statistical analysis of fluorescence units estimated in four experimental groups: vehicle control (C), SB-treated control (C + SB), vehicle-treated hypoxia-ischemia (HI), and SB-treated hypoxia-ischemia (HI + SB). SB application had no effect on the HI-induced activation of caspase-3 in the HI ipsilateral hemispheres. The values represent means ± SD from five animals. The one-way ANOVA and Bonferroni test: *p < 0.05, ***p < 0.001, ****p < 0.0001. *C* control, *ipsi* ipsilateral, *contra* contralateral

Activity of caspase-3 is expressed by the level of fluorescence generated upon cleavage of specific caspase-3 substrate (N-Ac-DEVD-N’-R110). As shown in the graph (Fig. 10), HI induced a significant increase in caspase-3 activity in the ipsilateral hemisphere noticed at 24 and 48 h of recovery, compared to sham control (*p* < 0.0001 and *p* < 0.05, respectively). Only a tendency to increase the activity of caspase-3 was simultaneously observed within the contralateral hypoxic hemisphere at 24 h after the insult. There was no effect of HDACi on the activity of caspase-3 in the injured ipsilateral side.

#### The assay of Bax and Bcl-2

**Fig. 11:**
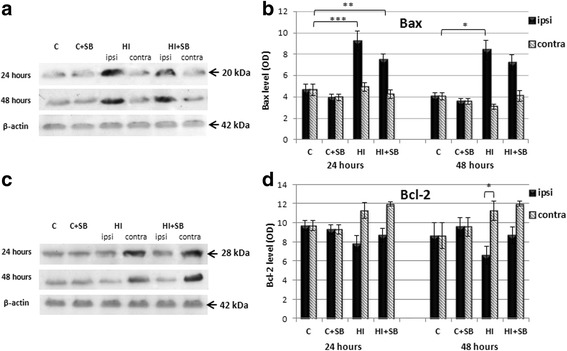
The effect of SB treatment on pro-apoptotic and anti-apoptotic protein levels in the brain after neonatal HI. Seven-day-old rats (PND7) were subjected to hypoxia-ischemia followed by 24 or 48 h of recovery. SB or vehicle was administered directly after the onset of HI and for 2 consecutive days. Figure shows representative immunoblots of pro-apoptotic Bax and anti-apoptotic Bcl-2 protein levels (**a**, **c**) in brain hemispheres, analyzed in four experimental groups: vehicle control (C), SB-treated control (C + SB), vehicle-treated hypoxia-ischemia (HI), and SB-treated hypoxia-ischemia (HI + SB). The intensity of each band obtained by Western blotting was quantified by LKB Ultrascan XL software and normalized in relation to β-actin. Bar graphs (**b**, **d**) represent statistical analysis of densitometric data from indicated experimental groups. Note the increased expression of Bax in the ipsilateral and Bcl-2 in the contralateral side after HI. The values represent means ± SD of five animals in each experimental group. The one-way ANOVA and Bonferroni test: *p < 0.05, **p < 0.01 and ***p < 0.001. *C* control, *ipsi* ipsilateral, *contra* contralateral

The levels of pro- and anti-apoptotic proteins Bax and Bcl-2, respectively, were evaluated by Western immunoblotting analyses. Figure 11 shows representative immunoblots and relative intensity of changes quantified by scanning densitometry.

Western blotting for Bax revealed that 24 h after HI, the level of this pro-apoptotic protein in the ipsilateral side increased about twofold compared to sham (*p* < 0.001). Higher than the control level of Bax expression was also observed at 48 h of recovery (*p* < 0.05). In contrast, at the same time, the expression of Bax protein in the contralateral hemisphere remained unchanged. SB injection had no apparent effect on the level of this protein in the brain; however, a tendency for it to decrease in relation to vehicle treatment was observed (Fig. 11a, b).

As it is shown in Fig. 11c, d, HI significantly increased the level of Bcl-2 in the contralateral, non-injured side pronounced at 48 h of recovery, compared to HI hemisphere (*p* < 0.05). Injection of SB after the injury did not change the HI-affected immunoreactivity.

## Discussion

The principal finding in our present study is that sodium butyrate treatment exhibits brain-protective activity in a neonatal hypoxia-ischemia model. The protection afforded by SB was expressed by a clear reduction of brain damage, suppression of brain edema, and preservation of brain architecture when analyzed at 6 days after the onset of hypoxia-ischemia. Furthermore, the effect of SB was associated with substantial inhibition of HI-induced inflammation. Our findings remain in general agreement with those reported previously that deacetylase inhibitors (VPA, TSA, SB) are neuroprotective in cerebral injury models in adult rodents [3–5]. Our data also agrees with a brief paper showing neuroprotection following treatment with valproate (VPA) after HI in neonatal rat [12].

Neonatal hypoxia-ischemia triggers a series of pathophysiological processes (including loss of energy, acidosis, excitotoxicity, elevation of intracellular calcium, induction of oxidative stress, inflammation) that result in a loss of neurons and severe neurological deficits. It is generally accepted that one of the most important pathogenic components of neonatal brain damage is inflammation induced by either the production of cytokines and chemokines followed by leukocyte (including monocytes and macrophages) infiltration or glial activation and proliferation [21–23]. First of all, it is in agreement that blocking the inflammatory reaction promotes neuroprotection and, in addition, has potential for use in the clinical treatment of ischemic brain injury [21, 24, 25].

Convincing evidence reveals that HDACis, among VPA, TSA and SB, are efficacious neuroprotective agents in adult cerebral injury models associated with inflammation. Administration of these compounds after the onset of stroke results in a marked reduction of microglia number, suppression of their activation, and inhibition of other inflammatory markers, which in turn lead to improved neuropathological outcome [4, 5]. In contrast to these findings, our results show that SB treatment of neonatal HI induced a paradoxical significant increase in the number of ED1-positive cells (microglia/macrophages) in the damaged ipsilateral hemisphere at 6 days after the insult, as compared to animals treated with vehicle. As demonstrated in the current study, the majority of ED-1+ cells present a positive reaction with an established marker of M2 microglia phenotype, arginase-1, mostly pronounced in the SB-treated rats. It may be speculated that SB facilitates conversion of M1 to M2 leading to anti-inflammatory signalling and, by this, keeps microglia from acquiring a proinflammatory phenotype, and in consequence prevents tissue damage, such as that found in models of AD, MS, and neurodegeneration [26–28]. This prediction may be reinforced by the parallel decrease in the number of ED-1/IL-1β positive cells observed in our study. The reduced cytokine response after SB treatment, despite an increase in the number of microglia, implies that these cells are not necessarily damaging and in some conditions may alleviate harmful consequences of injury. This hypothesis remains in line with data showing that transition in the microglial response during recovery from the proinflammatory (M1) to immunomodulatory and neurotrophic response (M2) [29–32] and then maintenance of endogenous neurogenesis [33–36] may play a key role in attenuation of brain damage [37]. To confirm the role of the microglial reaction to HI injury in the developing brain and, in particular, to define the time course of M1 to M2 polarization, further studies will be needed.

It is commonly known that reactive astrocytosis also appears to be a part of the hypoxia-ischemia-induced pathological processes [38, 39]. Consistent with previous reports [30, 40, 41], we noted a delayed increase in GFAP expression accompanied with hypertrophy and cell proliferation in the ipsilateral hemisphere at 6 days after the insult, implying astrogliosis. The expression of GFAP was further markedly increased by SB treatment. However, this increase was associated with the reduction of cell population co-expressing GFAP and proinflammatory IL-1β. It is worthy to note that SB treatment also led to diminished IL-β production in microglia/macrophages at the same time point. This reduction of IL-1β expression after SB injection in glial cells parallels the attenuation of brain damage. The precise molecular mechanism responsible for the effect of SB is not known. However, apart from the number of biochemical and morphological factors functioning in concert to influence the final SB effect, accumulation of GFAP protein presented here is likely to also contribute to neuroprotection after neonatal HI. This may be supported by data showing that GFAP knock-out mice have a greater susceptibility to ischemic injury [42]. Furthermore, experimental disruption of astroglial scar formation following stroke results in an increased spread of inflammation and increased lesion volume [43]. Although results obtained from adult experiments cannot be directly transferred and used as explanation for neonatal data due to differences in the level of maturation and different ischemia model, some hypotheses may be valid in adults as well as in neonates. Nevertheless, a precise role of enhanced astrogliosis seen after SB treatment of neonatal HI is yet to be determined.

Cytokines are regarded as pro- or anti-inflammatory, and based on their state and/or concentration, they can be protective or harmful. Although these proteins can be found in almost any nucleated cell within the brain, such as brain endothelial cells or neurons, they are mainly produced by glial cells or by immune cells, such as helper T cells. Therefore, in the present study, we followed the influence of SB administration on the total content of selected cytokines correlating with the brain damage. The biological effect of these factors include stimulation and synthesis of other cytokines and prompting leukocyte infiltration, which in turn leads to the induction of neuronal injury mediators and influencing glial expression (see rev [44]). Our results, in general accordance with other reports [40], depicted a considerable alteration in the expression of IL-1α, IL-1β, TNFα, and chemokine CXCL10 in the ipsilateral hemisphere at 48 h after HI compared to the control one. In line with this, we also observed a significant enhancement in IL-1β and TNFα mRNA level estimated 12 h following the insult. In addition to these early modifications, IL-1β and chemokine CXCL10 protein expression presented a delayed increase after 6 days of recovery suggesting ongoing inflammation. This is in agreement with reported elevation in mRNA and protein level of IL-1β even at 14 days after HI [45, 46]. Treatment with SB suppressed significantly HI-induced upregulation of chemokine CXCL10 at 48 h and IL-1β at 6 days after HI. In the case of IL-1α and TNFα, the effect of SB was presented only by a non-significant decrease in their level 48 h after the insult, despite a sole, clear reduction in TNFα mRNA expression in the same condition. Probably both factors do not play a prominent role in the protective action mediated by this inhibitor.

The reduction of IL-1β expression presented in our study seems to be particularly important and strongly supported by a number of data showing that downregulation of this cytokine plays a neuroprotective function in the development of HI encephalopathy [22, 29, 47, 48]. According to research, the decrease of IL-1β production can reverse cell swelling, brain edema, and neurologic function deficiencies induced by HI [49].

Despite the number of reports focusing on the role of IL-1β, only a few data are available on the potential role of chemokines in the development of HIE [50]. It was found in a neonatal mouse study of HI injury that mRNA expression of chemokines precedes infiltration of immune cells into the brain, thus proving their relevance in the inflammatory response. It is therefore reasonable to speculate that the reduction of CXCL10 expression observed in the present study participates, at least partially, in the beneficial action of SB. On the other hand, chemokines attract mesenchymal stem cells to home at the lesion site [51]. Hence, immunomodulatory intervention may have a negative effect upon specific aspects of neurogenesis and thus brain regeneration. Therefore, the question arises if the protective abilities will outweigh the potentially harmful consequences.

It has been suggested that in terms of anti-inflammatory effects, inhibition of COX-2 and subsequent reduction of prostaglandin E2 (PGE2) generation, a major downstream product of COX-2 enzymatic activity, can lead to attenuation of ischemic injury in adult rodents [52–54]. As demonstrated in the current study, SB administration decreased the HI-induced elevated COX-2 expression in the damaged ipsilateral hemisphere. This observation may be related to the reduced level of pro-inflammatory IL-1β at the same time point, as demonstrated by Neeb et al. [55]. Unexpectedly, the decreased expression of COX-2 after SB treatment seen 6 days post-HI does not result in diminished generation of PGE2. Moreover, the fact that COX-2 and PGE2 levels do not correlate in animal models of induced inflammation is also an interesting finding [56]. The lack of this correlation suggests that not COX-2 but COX-1 isoform may be expressed and be responsible for maintaining the PGE2 production under brain ischemia [54, 57–59]. Nevertheless, the reason for SB suppression of COX-2 and not PGE2 level in our study is unclear at present and should be explored in the future. Particular attention should be paid to the complexity of enzymatic pathways embedded in PGE2 synthesis and degradation, rather than focusing only on COX-1 and COX-2 concentration. In this context, it is noteworthy that PGE2 under defined conditions may not only contribute to brain damage but rather affect and modulate neuronal function in a positive way through the regulation of microcirculation and synaptic functions [60, 61].

Several findings indicate that inhibitors of histone deacetylases may also modify diverse targets including, among others, transcription factors such as NFκB and p 53, the HSP family of proteins, and apoptosis-related genes [4, 62, 63].

A number of reports point to the damaging role of activated by brain ischemia nuclear factor NFκB. This is supported by studies showing that inhibition of NFκB activation after ischemia in adult rodents prevents brain damage in the insulted hemisphere via inhibition of cytokine response [64–67]. However, our findings revealed that following neonatal hypoxia-ischemia, the expression of NFκB increased significantly in both hemispheres, ipsi- and contralateral, despite tissue alterations not being observed in the hypoxic, uninjured side. Moreover, in both hemispheres, the level of NFκB returned to the control value after SB treatment. Thus, the question arises whether the response of NfκB to SB may constitute part of the defense process against HI-induced damage in the ipsilateral side. It is worthy to mention that probably the basal level of NFκB is sufficient for conditions required for neonatal brain development.

An additional suggested factor by which HDACis are reported to mediate neuroprotection in adult cerebral injury models includes HSP70 [68–71]. HSP70 besides functioning as a key member of molecular chaperon system has also been assigned an anti-apoptotic function, although failure to detect protection against apoptosis in neurons overexpressing HSP70 also has been reported [72]. Nevertheless, most studies describe increased expression of HSP70 as a neuroprotective mechanism in adult rodents after MCAO [3, 73, 74], as well as after neonatal HI [75]. The suggested influence of HSP70 action includes inhibition of nuclear transcription factor—NFκB. In contrast to high expression of HSP70 at 12–48 h found by Van den Tweel [75] in the damaged HI hemisphere, our present results show significant reduction of this protein level at the same time point regardless of exposure to SB. Additionally, the changes in HSP70 expression observed in our studies do not parallel alterations seen in the level of NfκB. The major difference with our study is that we used P7 vs P12 rats and a different time of hypoxia—60 vs 90 min of hypoxia insult used by Van den Tweel [75]. The reason for the loss of HSP70 may be due to a low rate of its synthesis or increased activity of proteases able to digest HSP70. Also, our results are more clearly in agreement with Sun et al. [76], showing that HSP70 is only slightly altered, if at all, in P7 neurons after HI. Interestingly, SB treatment caused elevation of HSP70 expression in both brain hemispheres 6 days post-HI. It seems that such delayed response detected in both hemispheres has to be insult independent. It may be also considered that induction of HSP70 after SB treatment may facilitate neuroplasticity during recovery time and improve learning processes [77].

We also tested if SB-induced neuroprotection in the HI neonatal brain involves changes in the expression of p53-apoptosis regulating transcription factor. The implication that p53 plays a role in the response that follows a hypoxic-ischemic insult stems from the observation that pifithrin alpha, an inhibitor of p53, decreases the number of apoptotic cells in the ischemic brain [78]. In contrast to the robust upregulation of p53 detected in the adult ischemia model in rodents and inhibition of p53 protein levels by SB [4], HI induced in neonates with/or without SB treatment did not show any significant effect. Thus, p53 seems to not contribute to the protective effect of SB.

Finally, our results revealed no apparent effect on caspase-3 activation, as well as on expression of anti-apoptotic proteins Bcl-2 and pro-apoptotic Bax. Therefore, these targets probably do not mediate SB-induced neuroprotection.

## Conclusions

In conclusion, we demonstrated that SB, an inhibitor of histone deacetylases, has significant neuroprotective abilities in a model of HI-induced neonatal brain injury. However, we were incapable of finding the precise mechanism by which SB exerts its actions. The mechanisms associated with the outcome of SB were not in agreement with those reported in adult cerebral injury studies. Nevertheless, the present results imply that some effects may be mediated by suppression of inflammation. Based on the findings obtained in our laboratory [10], it is also tempting to speculate that the delayed neuroprotective action may be mediated in part by increased proliferation and/or neurogenesis.

## Abbreviations

AD: Alzheimer’s disease
ANOVA: Analysis of variance
Arg-1: Arginase 1
Bcl-2: B-cell lymphoma 2
C: Control
CONTRA: Contralateral hemisphere
COX-2: Cyclooxygenase-2
CXCL10 (IP-10): C-X-C motif chemokine 10
ED1 (CD68): Cluster of Differentiation 68
ELISA: Enzyme-linked immunosorbent assay
GFAP: Glial fibrillary acidic protein
HDACi: Histone deacetylase inhibitor
HE: Hematoxylin and eosin stain
HI: Hypoxia-ischemia
HSP70: Heat shock protein 70
IFN-γ: Interferon gamma
IL-12: Interleukin 12
IL-1α: Interleukin 1 alpha
IL-1β: Interleukin 1 beta
IL-2: Interleukin 2
IL-4: Interleukin 4
IPSI: Ipsilateral hemisphere
MCAO: Middle cerebral artery occlusion
NFκB: Nuclear factor kappa-light-chain-enhancer of activated B cells
PBS: Phosphate-buffered saline
PFA: Paraformaldehyde
PGE2: Prostaglandin E2
PMSF: Phenylmethylsulfonyl fluoride
PND7: Postnatal day 7
qRT-PCR: Quantitative reverse transcriptase real-time polymerase chain reaction
SAHA: Suberoylanilide hydroxamic acid
SB: Sodium butyrate
SDHA: Succinate dehydrogenase complex flavoprotein subunit A
SDS-PAGE: Sodium dodecyl sulfate polyacrylamide gel electrophoresis
TNFα: Tumor necrosis factor alpha
TSA: Trichostatin A
VPA: Valproate
